# Prevention and Mitigation of Acute Radiation Syndrome in Mice by Synthetic Lipopeptide Agonists of Toll-Like Receptor 2 (TLR2)

**DOI:** 10.1371/journal.pone.0033044

**Published:** 2012-03-27

**Authors:** Alexander N. Shakhov, Vijay K. Singh, Frederick Bone, Alec Cheney, Yevgeniy Kononov, Peter Krasnov, Troitza K. Bratanova-Toshkova, Vera V. Shakhova, Jason Young, Michael M. Weil, Angela Panoskaltsis-Mortari, Christie M. Orschell, Patricia S. Baker, Andrei Gudkov, Elena Feinstein

**Affiliations:** 1 Cleveland BioLabs, Inc., Buffalo, New York, United States of America; 2 Radiation Countermeasures Program, Armed Forces Radiobiology Research Institute, Uniformed Services University of the Health Sciences, Bethesda, Maryland, United States of America; 3 Department of Radiation Biology, F. Edward Hébert School of Medicine, Uniformed Services University of the Health Sciences, Bethesda, Maryland, United States of America; 4 Department of Environmental and Radiological Health Sciences, Colorado State University, Fort Collins, Colorado, United States of America; 5 Division of Hematology, Oncology and Blood and Marrow Transplant, Department of Pediatrics, University of Minnesota, Minneapolis, Minnesota, United States of America; 6 Division of Pulmonary, Allergy and Critical Care Medicine, Department of Medicine, University of Minnesota, Minneapolis, Minnesota, United States of America; 7 Indiana University School of Medicine, Indianapolis, Indiana, United States of America; 8 Roswell Park Cancer Institute, Buffalo, New York, United States of America; Charité-University Medicine Berlin, Germany

## Abstract

Bacterial lipoproteins (BLP) induce innate immune responses in mammals by activating heterodimeric receptor complexes containing Toll-like receptor 2 (TLR2). TLR2 signaling results in nuclear factor-kappaB (NF-κB)-dependent upregulation of anti-apoptotic factors, anti-oxidants and cytokines, all of which have been implicated in radiation protection. Here we demonstrate that synthetic lipopeptides (sLP) that mimic the structure of naturally occurring mycoplasmal BLP significantly increase mouse survival following lethal total body irradiation (TBI) when administered between 48 hours before and 24 hours after irradiation. The TBI dose ranges against which sLP are effective indicate that sLP primarily impact the hematopoietic (HP) component of acute radiation syndrome. Indeed, sLP treatment accelerated recovery of bone marrow (BM) and spleen cellularity and ameliorated thrombocytopenia of irradiated mice. sLP did not improve survival of irradiated TLR2-knockout mice, confirming that sLP-mediated radioprotection requires TLR2. However, sLP was radioprotective in chimeric mice containing TLR2-null BM on a wild type background, indicating that radioprotection of the HP system by sLP is, at least in part, indirect and initiated in non-BM cells. sLP injection resulted in strong transient induction of multiple cytokines with known roles in hematopoiesis, including granulocyte colony-stimulating factor (G-CSF), keratinocyte chemoattractant (KC) and interleukin-6 (IL-6). sLP-induced cytokines, particularly G-CSF, are likely mediators of the radioprotective/mitigative activity of sLP. This study illustrates the strong potential of LP-based TLR2 agonists for anti-radiation prophylaxis and therapy in defense and medical scenarios.

## Introduction

Acute high dose radiation exposure affecting large populations could result from multiple potential disaster scenarios, thus dictating the need for safe and effective medical radiation countermeasures (MRC) [1], [2]. Use of MRC would be aimed at reducing near-term mortality as well as limiting radiation damage that causes long-term adverse health effects [3], [4]. In addition, large numbers of people are routinely exposed to anti-cancer radiation therapy and MRC could be useful in ameliorating the negative side effects of the therapy and enabling safe application of higher doses of radiation [5], [6].

The biological effects of radiation on mammalian organisms are strongly dependent upon the dose of radiation received [4]. Acute radiation syndrome (ARS) developing from whole-body or significant partial-body irradiation can involve hematopoietic (HP), gastrointestinal (GI), and cerebrovascular components [7]. Cerebrovascular damage caused by massive neuronal apoptosis is induced by the highest radiation doses (more than 10–20 Gy in humans) and invariably leads to death within several days. In contrast, mortality from HP syndrome (induced by TBI doses of 1 Gy or more in humans) and GI syndrome (induced by 5 Gy or more) occurs with lower frequency and more slowly (over weeks rather than days) and is more likely to be amenable to pharmacological countermeasures. Notably, GI syndrome always occurs in concert with HP syndrome, and the HP component can play a major role in mortality even if it stems primarily from GI damage. For example, death of patients with GI ARS is frequently caused by sepsis resulting from death of epithelial cells lining the GI tract and loss of GI tract integrity together with concomitant loss of immune/hematopoietic function. Even though isolated HP syndrome is induced by lower radiation doses and has a better prognosis than GI syndrome, it is a serious concern, typically resulting in 50% mortality within ∼3–6 weeks. Moreover, since HP syndrome occurs at the lowest ARS-inducing dose range of irradiation, it would likely affect the largest proportion of an exposed population.

There are a number of potential MRC currently at different stages of development, which fall roughly into two categories depending upon their primary mechanism of action: immunomodulators/cytokines/growth factors [8], [9], [10] and antioxidants/free radical scavengers [11], [12]. In large part, the focus on cytokines and growth factors has been based on their potential ability to act as radiomitigators enhancing recovery of the HP system from radiation damage, as demonstrated in multiple in vitro and in vivo models [8], [13]. These include stem cell factor (SCF) [14], [15], FMS-like tyrosine kinase-3 (FLT-3) ligand [16], [17], interleukin-1 fragment (IL-1β-rd) [18], keratinocyte growth factor (KGF) [19], and G-CSF [9]. Some cytokines have received FDA approval for treatment of neutropenia and thrombocytopenia caused by anti-cancer radiotherapy and chemotherapy, and several are in development [2], [3], [20]. The anti-radiation potential of antioxidants and free radical scavengers derives from their ability to reduce levels of reactive oxygen species (ROS) induced by radiation, thus decreasing DNA damage, lipid peroxidation and other types of chemical modification damage [12]. This category of anti-radiation drugs is represented by the naturally occurring antioxidant vitamin E and the synthetic phosphorothioate amifostine (WR-2721), which is currently used to minimize the side effects of radiation therapy [21]. Efficacy of this class of agents is limited to prophylaxis of radiation injury.

Since ARS involves massive apoptosis in radiosensitive tissues such as the HP system and GI tract [22], [23], [24], we have focused on developing novel strategies to reduce radiation damage and lethality by targeting cellular pathways that regulate apoptosis. One such pathway involves a major regulator of all aspects of immune responses, NF-κB, which mediates transcriptional upregulation of anti-apoptotic genes [25]. The survival-promoting capacity of this pathway is indicated by the fact that constitutive activation of NF-κB is a common feature of tumors [26]. We hypothesized that pharmacological activation of NF-κB might be a promising strategy for both radioprotection and radiomitigation since NF-κB regulates expression of not only anti-apoptotic genes [25], but also those encoding (i) cytokines and growth factors that induce proliferation and survival of HP and other stem cells [27], [28]; and (ii) potent ROS-scavenging antioxidant proteins, such as MnSOD (SOD-2) [29]. The rationale for exploring this approach was further strengthened by the finding that mice with a genetic defect in NF-κB signaling displayed heightened GI radiosensitivity [30].

To develop pharmacological activators of NF-κB, we exploited one of the natural mechanisms by which the innate immune system responds to microbial infections. Various pathogen-associated molecular patterns (PAMPs) are recognized by host cells due to their specific interaction with Toll-like receptors (TLRs), which leads to activation of NF-κB [31]. We hypothesized that drugs based on PAMPs might be safe since they are commonly present in humans [32]. These features, as well as significant in vivo radioprotective/mitigative efficacy, were demonstrated for an NF-κB-activating TLR5 agonist based on bacterial flagellin [33]. In the current work, we investigated use of another type of PAMP, TLR2/6 agonistic synthetic mycoplasma-derived lipoproteins (sLP), as radiation countermeasures.

Here we demonstrate that synthetic mimetics of diacylated mycoplasma lipopeptides (sLP; e.g., Pam_2_-CSKKKK), agonists of TLR2, have significant in vivo radioprotective and radiomitigative efficacy. A single injection of sLP given either before or after irradiation increased the survival of mice exposed to doses of total body irradiation (TBI) inducing mortality primarily from the HP component of ARS, but not those inducing GI-related ARS, and had beneficial effects on bone marrow and spleen cellularity and platelet levels. sLP injection led to strong induction of a number of cytokines with known roles in hematopoiesis, which likely contributes to the radioprotective/mitigative activity of sLP. Finally, through comparison of SLP-mediated cytokine induction and radiation protection in reciprocal chimeric mice with either wild type or TLR2-null bone marrow, we demonstrated that the ability of SLP to protect against radiation damage to the HP system is, at least in part, due to indirect effects of responses initiated in non-bone marrow-derived cells. Overall, this study provides a foundation for development of LP-based TLR2 agonists for anti-radiation prophylaxis and therapy in defense and medical scenarios.

## Results

### A synthetic mycoplasma lipopeptide mimetic (sLP) protects mice from radiation-induced death

To test our hypothesis that pharmacologic imitation of BLP-mediated TLR2 stimulation might be an effective anti-radiation strategy, we used a synthetic mimetic of a mycoplasma di-palmitoylated lipopeptide (R,R-Pam_2_Cys-SKKKK, sLP) as a TLR2 ligand. We chose to focus on LPs from mycoplasma rather than other types of bacteria since these organisms typically exist asymptomatically as part of the commensal microflora in mammals [32] and their products could, therefore, be expected to have low toxicity and immunogenicity. Moreover, sLP was previously shown to mimic the NF-κB-induction activity of natural *Mycoplasma*-derived lipoproteins [34], [35], [36], [37]. We confirmed that sLP activated an NF-κB-dependent LacZ reporter in cultured HEK293 cells expressing TLR2/6 or TLR2/CD14 receptors, but not in TLR2/1 expressing reporter cells or in TLR-negative HEK293 cells (data not shown).

**Figure 1:**
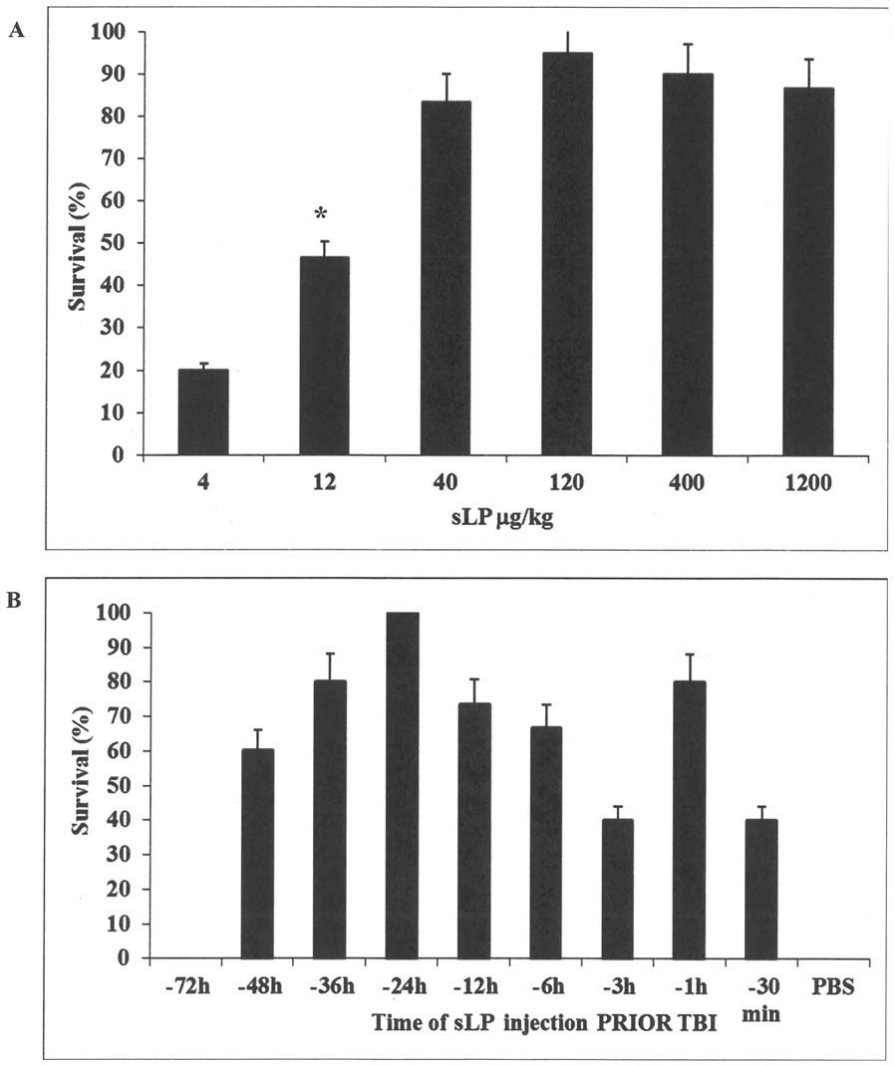
Synthetic lipopeptide (sLP) protects mice from radiation-induced death. (A) Dose-dependence of sLP-mediated radioprotection. Female ICR (CD-1®) mice were injected sc with sLP (4, 12, 40, 120, 400, or 1200 µg/kg; *n = 15*/group; average mouse weight 25±2 g) or PBS vehicle (*n = 10*). Mice were irradiated with 10 Gy TBI 24 h after injection and monitored for survival for 30 days. The average 30-day survival from 3 independent experiments is presented. Error bars indicate standard error. No animals in the PBS-injected control group survived to day 30. The differences in 30-day survival between sLP-treated and PBS-treated groups were statistically significant for sLP doses ≥12 µg/kg (as indicated by *, P<0.05, Fisher's Exact Test). (B) Time window for effective pre-irradiation administration of sLP. Female ICR mice (*n = 15*/group) were injected sc with sLP (40 µg/kg) at the indicated time points before irradiation with 10 Gy TBI (30 min, 1, 3, 6, 12, 24, 36, 48, or 72 h before TBI at time “0”). The control group (*n* = 15) was injected with PBS 24 h before 10 Gy TBI. The average 30-day survival in each group in 2 independent experiments is shown. Error bars indicate standard error. Injection of sLP at all times except for −72 h resulted in statistically significant improvement in 30-day survival relative to PBS treatment (P<0.05, Fisher's Exact test). (C) Determination of the dose reduction factor (DRF) for the optimal radioprotective regimen of sLP administration. Probit analysis was performed using Kaplan-Meier survival curves generated from treatment of female ICR mice with PBS vehicle or 40 µg/kg sLP at 24 h prior to exposure to different TBI doses (see figure S1).

Groups of female ICR mice (n = 15/group) were injected subcutaneously (sc) with a wide range of doses of sLP followed by exposure to 10 Gy total body gamma-irradiation (TBI) 24 hours (h) after sLP injection, and were then monitored for survival for 30 days. 10 Gy is equivalent to an LD_100/30_ dose in ICR mice, resulting in death of 100% of mice within 30 days (Figure 1). As shown in Figure 1A, pretreatment with a single dose of sLP dramatically improved the survival of mice exposed to this otherwise lethal dose of TBI. The survival benefit was dose-dependent. A substantial, although not statistically significant, increase in survival was observed even with the lowest tested dose of 4 µg/kg sLP (20% survival as compared to 0% in the PBS-injected control group, *p* = 0.25). Survival increased to 46% with 12 µg/kg sLP and 82% with 40 µg/kg sLP (*p* = 0.02 and 0.0001, respectively, for comparison to the PBS-treated group). Doses of 40–1200 µg/kg resulted in similar levels of radioprotection (82–95% survival, *p*<0.05 for comparison to the PBS-treated group, *p*>0.05 for all pair-wise comparisons between different sLP dose groups). Based on these results, 40 µg/kg was selected as the optimal radioprotective dose of sLP for use in subsequent experiments. Specificity of the radioprotective effect of sLP mimicking *Mycoplasma*-derived lipopeptides was indicated by our finding that (Pam_3_)-CSKKKK, a synthetic lipopeptide mimicking bacterial-derived that lacks the lipid moiety typical of *Mycoplasma*-derived lipopeptides and does not activate NF-κB-dependent reporter expression *in vitro*, did not improve survival of irradiated mice (data not shown).

In order to determine the time window for effective administration of sLP relative to radiation exposure, we injected female ICR mice sc with 40 µg/kg sLP at different time points prior to 10 Gy TBI. The 30-day survival rate was 0% for both the control group injected with PBS at 24 h prior to TBI and the group injected with sLP at 72 h prior to TBI (Figure 1B). However, injection of sLP at any of the other tested times (48, 36, 24, 12, 6, 3, 1 and 0.5 h prior to TBI) resulted in a significant increase in 30-day survival relative to PBS injection (40–100% survival, *p* = 0.02 for comparison of sLP-treated groups with the lowest 30-day survival (40%) as compared to the PBS-treated group). Injection of sLP 24 h prior to TBI resulted in 100% survival and was therefore selected as the optimal radioprotective time of administration. It is notable, however, that the effective time window for achieving significant radioprotection with sLP is broad, ranging from 48 h to 0.5 h prior to irradiation.

Having established an optimal radioprotective sLP dosing regimen of 40 µg/kg injected sc 24 h before TBI, we tested this regimen (versus PBS vehicle injection as a negative control) against a range of TBI doses (Figure S1). Coverage of LD_0–100/30_ doses of TBI required ranges of 7–10 Gy for the vehicle-treated groups (Figure S1A) or 10–13 Gy for the sLP-treated groups (Figure S1B). The LD_50/30_ was found to be 8.5 Gy for vehicle-treated mice and 11.25 Gy for sLP-treated mice using probit analysis (see below). Thus, it is clear that sLP allows mice to survive exposure to higher radiation doses than vehicle. However, there was a limit to the level of TBI against which sLP was able to protect a significant number of mice. While sLP provided complete protection of mice from 10 Gy TBI (100% 30-day survival), its efficacy was lower at higher TBI doses. Thirty-day survival in sLP-treated groups exposed to 10.5, 11.0, 11.5, 12.0, and 13.0 Gy TBI was 87%, 63%, 28%, 13%, and 0%, respectively (Fig. S1B). Given the well-defined preference of HP-ARS or GI-ARS-induced mortality by different levels of radiation exposure [4], the dramatic difference in the ability of sLP to improve survival of ICR mice irradiated with 10 Gy versus 12 Gy suggests that this agent is effective only against HP-arm of ARS.

The survival curves established for vehicle and sLP treatment at different TBI doses (Figure S1A,B) were used to determine the dose reduction factor (DRF) for sLP using probit analysis (Figure 1C). DRF, defined as the ratio of LD_50/30_ between drug- and vehicle-treated groups, is a standard means for quantifying the radioprotective efficacy of drugs. This analysis revealed that sLP increased the LD_50/30_ in the ICR mouse model from 8.5 Gy to 11.25 Gy, yielding a DRF of approximately 1.32. The slopes of the probit lines for the drug- and vehicle-treated groups were not substantially different; however, the probit line for the drug-treated groups was shifted significantly to the right (*p*<0.05).

### sLP acts as a radiomitigator to improve mouse survival when administered after irradiation

**Figure 2:**
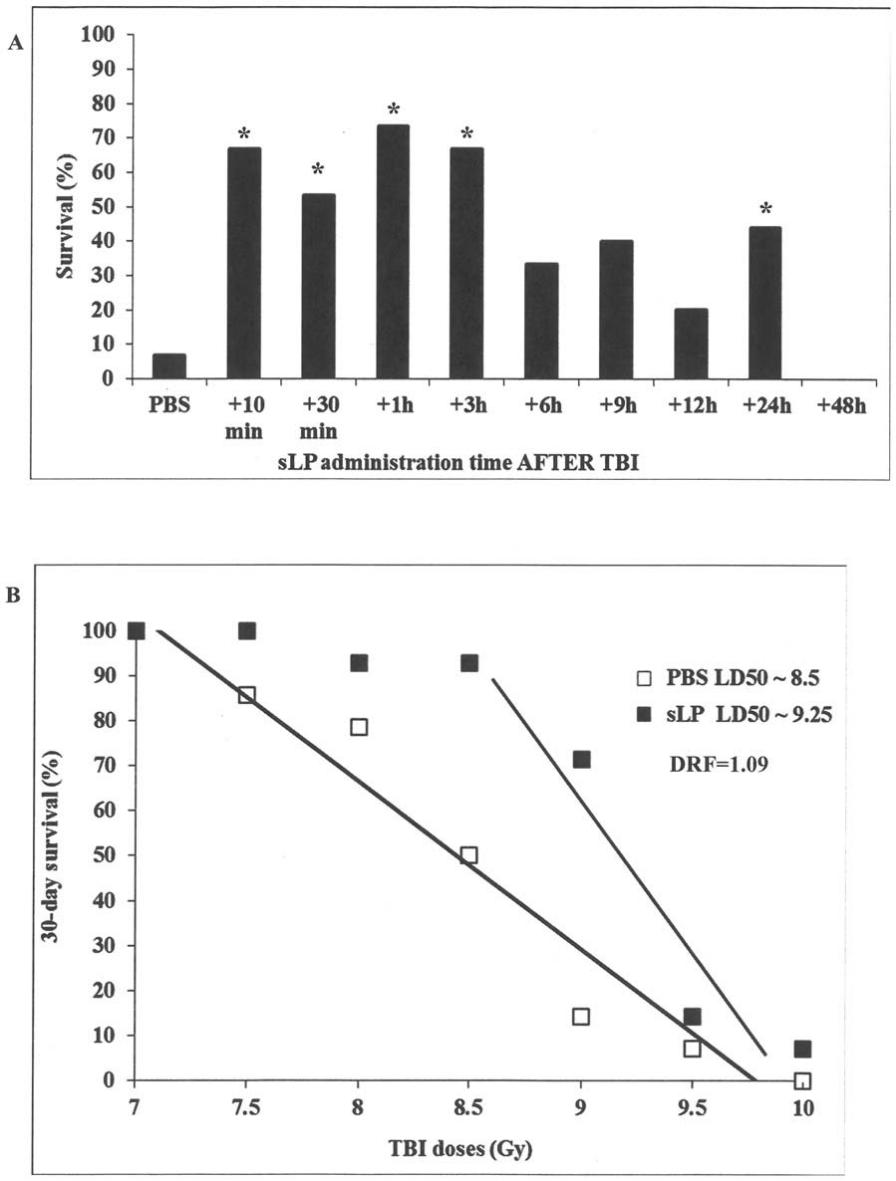
Radiomitigation by sLP: administration of sLP after lethal TBI improves mouse survival. (A) Time window for effective post-irradiation administration of sLP. Female ICR (CD-1®) mice (*n = 15*/group) were irradiated with 9 Gy TBI (at time “0”) and then injected sc with PBS at 1 h after irradiation or with sLP (50 µg/mouse) at 10 or 30 min, or 1, 3, 6, 9, 12, 24, or 48 h after irradiation. Mouse survival was monitored for 30 days. The differences in 30-day survival between the vehicle-treated group and groups treated with sLP between 10 min and 3 h after TBI and at 24 h after TBI were statistically significant (as indicated by *, P<0.05, Fisher's Exact test). (B) Determination of the DRF for the optimal radiomitigative regimen of sLP administration. Probit analysis was performed using Kaplan-Meier survival curves generated from treatment of female ICR mice with PBS vehicle or 50 µg/mouse sLP at 1 h after exposure to different TBI doses (see figure S2A). (C) SLP-mediated mitigation of death induced by low dose rate irradiation. Female ICR (CD-1®) mice (*n = 15*/group) received TBI at a continuous exposure rate of 0.4 cGy/min for 60 or 70 h (total dose of 14.4 or 16.8 Gy, respectively). At the mid-point of the irradiation period (i.e., 30 or 35 h after irradiation was started for the 60 and 70 h TBI groups, respectively), irradiation was halted for about 5 min during which time mice were injected sc with 10 µg/mouse sLP. Mouse survival was monitored for 30 days.

Having demonstrated significant radioprotective efficacy for sLP administered prior to radiation exposure, we evaluated whether sLP could also act as a radiomitigator and improve mouse survival when injected *after* irradiation. Female ICR mice were injected sc with sLP or PBS at different times after TBI and monitored for survival. sLP did not improve 30-day survival when administered to mice at any time point after exposure to TBI doses of 10 Gy (LD_100/30_) or greater. However, at lower TBI doses (e.g., LD_50/30_–LD_90/30_, 8.5–9 Gy), sLP treatment led to significantly improved 30-day survival as compared to vehicle treatment (data not shown). For mice exposed to 9 Gy TBI, injection of sLP at all tested times of administration from 10 min to 24 h post-irradiation increased 30-day survival relative to PBS injection (Figure 2A). Injection of sLP 48 h after 9 Gy TBI did not have any beneficial effect on mouse survival. The observed sLP-mediated survival benefit was statistically significant for administration at 10 and 30 min and 1, 3, and 24 h post-irradiation (*p*<0.05), but not for administration at 6, 9, or 12 h post-irradiation. Thus, sLP showed significant radiomitigative efficacy when injected as a single dose as late as 24 h after radiation exposure. However, since the greatest degree of radiomitigation was observed with injection of sLP 1 h after TBI (73% survival versus 7% in the vehicle-treated group, *p* = 0.0002), this time point was selected as the optimal time point for radiomitigation by sLP.

The DRF for post-irradiation administration of sLP was determined using probit analysis as described above for the pre-irradiation regimen of sLP treatment. Thirty-day survival curves were established for groups of female ICR mice injected with 50 µg/mouse sLP 1 h after exposure to 7.5, 8.0, 8.5, 9.0, 9.5, or 10 Gy TBI (Figure S2A). The LD_50/30_ for this regimen of sLP treatment was found to be approximately 9.25 Gy as compared to 8.5 Gy for vehicle treatment, indicating a DRF of 1.09 (Figure 2B). This is illustrated by the slight shift in the probit line for sLP treatment to the right as compared to the line for vehicle treatment; however, the difference between the drug- and vehicle-treated groups was not significant (*p*>0.05). The different slopes of the probit lines for drug- and vehicle-treated groups is illustrative of the lack of sLP-mediated radiomitigation at higher TBI doses (e.g., 9.5–10 Gy) as compared to lower doses (e.g., 8.5–9.0 Gy).

Despite its relatively low DRF for radiomitigation, it is clear that following certain TBI doses, sLP injection effectively mitigates radiation-induced death (Figure 2, S2A). In fact, we found that a single dose of 50 µg/mouse sLP given 3 h after 8 Gy TBI was as effective in rescuing C57BL/6 mice as 16 daily post-irradiation injections of recombinant G-CSF (Neupogen®, Amgen, Inc.), the current standard of care for myelosuppression associated with cancer treatment (Figure S2B). Survival was increased from 70% (21/30) in the PBS-treated group to 96.7% (29/30) and 96.2% (25/26) in the sLP at +3 h and G-CSF×16 treatment groups, respectively. When given as late as 24 h post-TBI, the single dose of 50 µg/mouse sLP increased survival to 90% (27/30). The increases in survival afforded by sLP at +3 h and G-CSF were statistically significant relative to the PBS control (*p* = 0.006 and *p* = 0.014, respectively), while survival increase by injection of sLP at +24 h was marginally significant compared to PBS (*p* = 0.054). There were no statistically significant differences in survival between any of the treated groups in this experiment.

### sLP is efficacious in the context of low dose rate total body irradiation

The experiments described above, as well as the majority of those reported in the radiobiology field, were performed with equipment that delivers radiation at a high dose rate (0.1–1 Gy/min), such that LD_30–100/30_ doses are achieved within a matter of minutes. While the radiomitigative efficacy of sLP under such conditions is important, we also wished to evaluate the radiomitigative potential of sLP under another realistic scenario of irradiation that involves high dose radiation received at a low dose rate. We first established that, in female ICR mice, delivery of approximate LD_50_ and LD_90_ doses of TBI at a rate of 0.4 cGy/min required 60 and 70 h of continuous irradiation, respectively (data not shown). Assessment of the morphology of the small intestine in morbid animals following irradiation under these conditions indicated that gastrointestinal (GI) damage was insignificant; therefore, damage to the HP system is likely to play a primary role in the lethality of these low dose rate irradiation regimens (data not shown). We next performed a survival experiment in which groups of mice were irradiated at 0.4 cGy/min for 60 or 70 h and injected sc with either PBS or 10 µg/mouse sLP half-way through the irradiation period (i.e., at 30 or 35 h after the start of irradiation). As shown in figure 2C, under both scenarios of “low dose rate” TBI, injection of sLP significantly improved 30-day survival of mice. With 60 h of irradiation (14.4 Gy TBI), 30-day survival was 100% (15/15) in the sLP-treated group as compared to 40% (6/15) in the PBS-treated group. Administration of sLP at the mid-point of 70 h of irradiation (16.8 Gy TBI) increased survival from 20% (3/15) in the PBS-treated group to 80% (12/15) in the sLP-treated group. For both TBI doses, the survival benefit provided by sLP treatment was statistically significant (*p*<0.05).

### sLP treatment promotes recovery of the hematopoietic system following irradiation

As described above, the TBI dose range against which sLP demonstrated radioprotective efficacy suggested that the drug is specifically effective in protecting and/or promoting regeneration of cells and tissues of the HP system (as opposed to cells of the GI tract). In addition, mice rescued from radiation-induced death by sLP treatment survived for at least 9 months with no evidence of hematopoietic failure and showing normal structure and cellularity of hematopoietic and lymphoid organs (data not shown). This indicates that sLP induced long-term repopulation/recovery of the HP system. The beneficial effect of sLP on the HP system in irradiated animals was directly demonstrated by our finding that sLP pretreatment accelerated recovery of cellularity of both the bone marrow and spleen in BALB/c mice exposed to a sub-lethal dose of 4 Gy TBI (Figure S3). Moreover, we showed that sLP reduced the severity of radiation-induced thrombocytopenia and accelerated recovery of circulating platelet levels (data not shown). This is a highly relevant finding since the severity of radiation-induced thrombocytopenia has been identified as the HP marker that is most closely correlated with radiation-induced mortality in primates [38].

### Administration of sLP leads to induction of multiple cytokines with potential roles in radioprotection and recovery of the hematopoietic system

**Figure 3:**
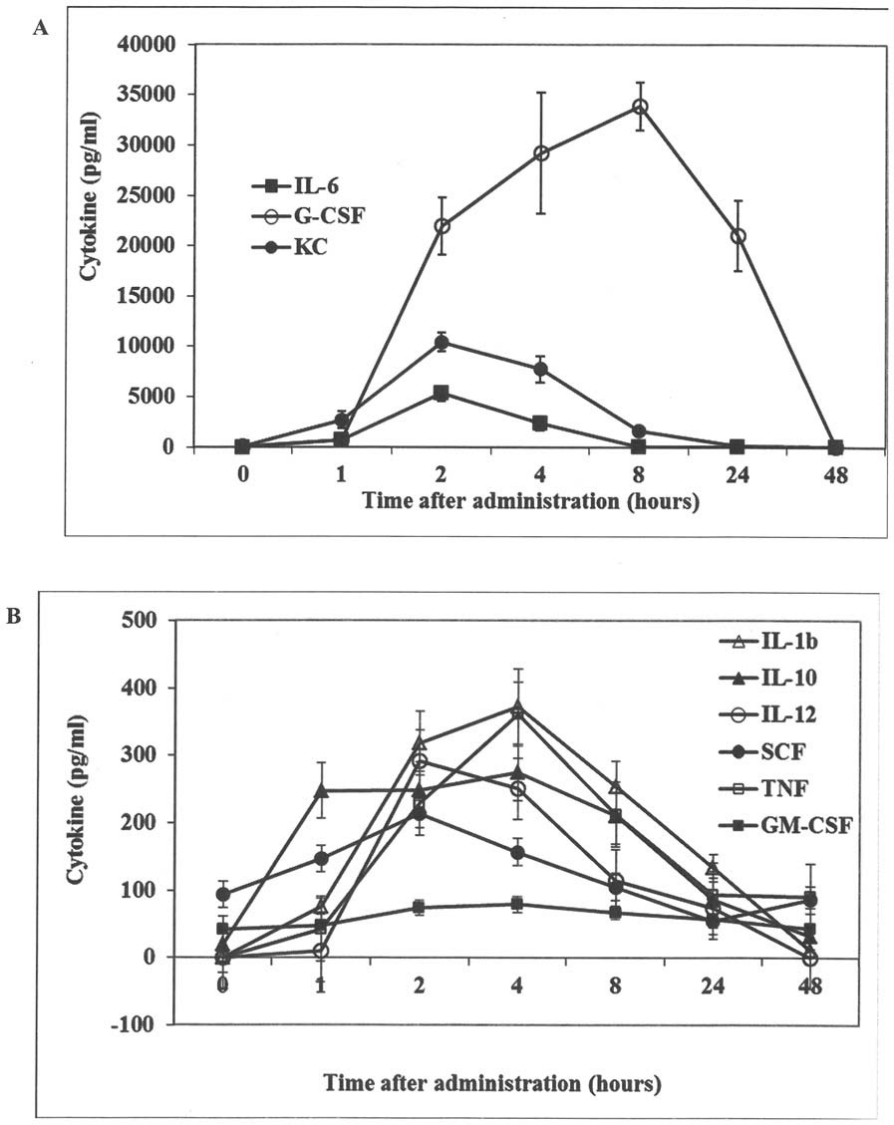
Administration of sLP in mice results in cytokine induction. Non-irradiated female C57BL/6 mice were injected sc with 10 µg sLP and euthanized 1, 2, 4, 8, 24, or 48 h later (*n* = 6/time point) for blood collection. Blood from untreated female C57BL/6 mice (*n* = 6) was analyzed as the “0 hour” time point. G-CSF, IL-6, and KC (A) and IL-1β, IL-10, IL-12(p70), SCF, GM-CSF, and TNF-α (B) levels were determined in individual mouse serum samples using multiplex Luminex assays. The mean cytokine concentration at each time point is shown. Error bars indicate standard error.

In order to gain insight into the mechanism(s) underlying the radioprotective/mitigative activity of sLP, we assessed whether sLP treatment of mice affected their serum levels of various cytokines. Our rationale for testing cytokine levels was three-fold: (i) sLP counteracted HP syndrome and cytokines are known to regulate survival and proliferation of HP cells, (ii) many cytokine-encoding genes are transcriptional targets of NF-κB, which is activated downstream of sLP-TLR interaction, and (iii) a number of cytokines were previously shown to have radioprotective efficacy [8]. Therefore, we collected blood samples from groups of 6 female C57BL/6 mice per time point at 1, 2, 4, 8, 24, and 48 h after sc injection of 10 µg/mouse sLP (without irradiation). Blood samples from 6 untreated female C57BL/6 mice were collected for use as a baseline control (referred to as time “0”). Analysis of blood serum samples in multiplex Luminex assays provided quantitation of G-CSF, IL-6, KC, IL-1β, IL-10, IL-12(p70), SCF, granulocyte macrophage colony-stimulating factor (GM-CSF), and tumor necrosis factor (TNF) levels (Figure 3A,B). All of the tested cytokines showed transient induction following sLP administration, but the extent of induction was much higher for IL-6, G-CSF and KC than for the other induced cytokines. Peak levels of IL-6, G-CSF, and KC were 34,000-, 10,000- and 5,000-fold higher than baseline, respectively. In contrast, peak levels of other induced cytokines were in the range of 50- to 375-fold higher than baseline. Levels of IL-6, G-CSF and KC were increased at the earliest tested time point (1 h post-injection) and, for IL-6 and KC, peaked 2 h after injection and returned to baseline within 8–24 h post-injection. Induction of G-CSF by sLP was unique among the tested cytokines both in the strength of the response (fold increase over baseline) and its duration. G-CSF levels peaked at 8 h post-injection, but high levels (more than 20,000-fold over baseline) persisted from 2 h to 24 h post-injection, only returning to baseline at 48 h after injection. As shown in Figure 3B, IL-1β, IL-10, IL-12(p70), SCF and TNF also showed induction within the first several hours after sLP administration, peaking at 2–4 h post-injection and declining to baseline within 24–72 h. GM-CSF was distinct in that it showed very moderate induction as compared to the other tested cytokines. These results demonstrate that sLP treatment induces multiple cytokines and suggests that activity of the induced cytokines may underlie, at least in part, the radioprotective/mitigative effects of sLP. The list of cytokines that were measured but did not change in response to sLP administration includes TPO, IL1α, IFN-γ, IL-2, IL-3, IL-4, and IL-11(data not shown).

### The radioprotective/mitigative activity of sLP requires TLR2 signaling

**Figure 4:**
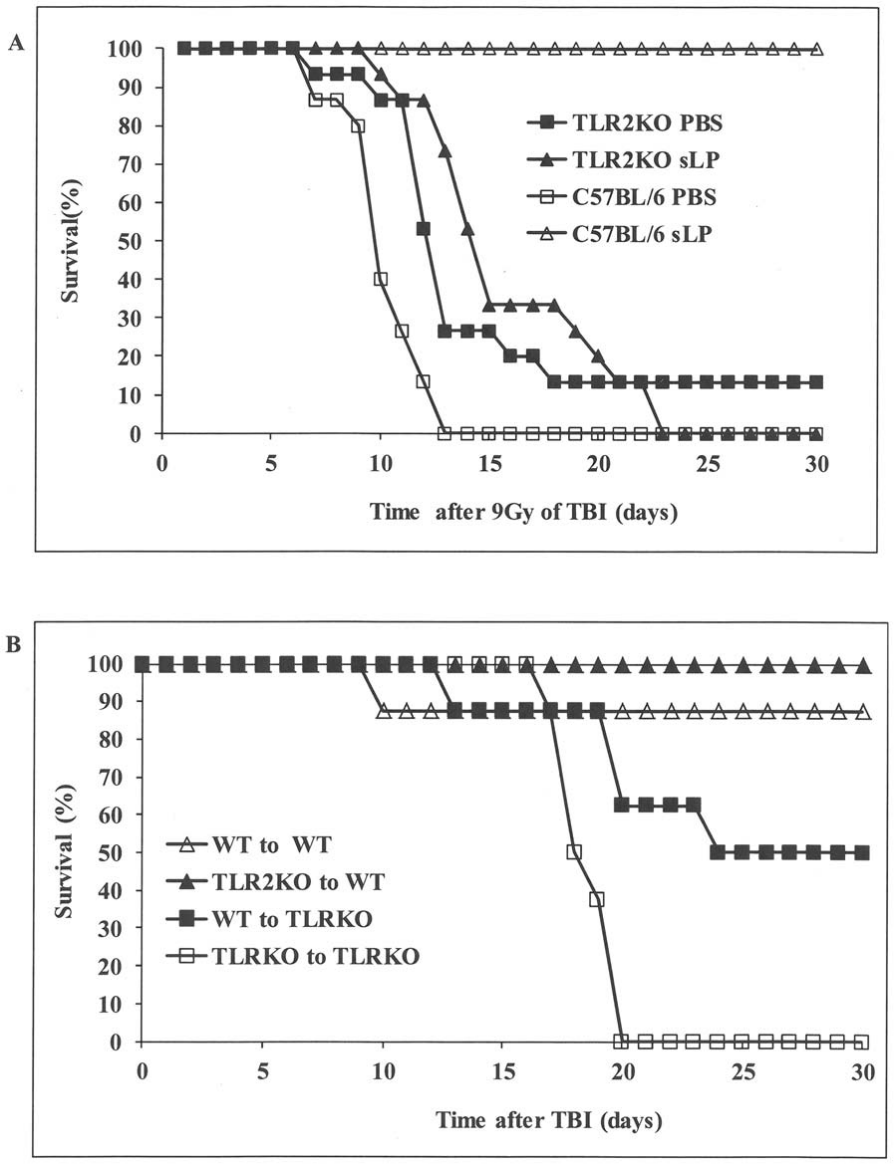
sLP-mediated radioprotection and cytokine induction is TLR2-dependent and involves both bone marrow and non-bone marrow cells. (A) TLR2-dependence of sLP-mediated radioprotection. Groups of isogenic TLR2(−/−) and wild type C57BL/6 mice (*n = 15*/group) were injected sc with vehicle (PBS) or sLP (3 µg/mouse) and irradiated (9 Gy TBI) 24 h later. Survival was monitored for 30 days. (B) sLP-mediated radioprotection in chimeric mice with wild type (WT) or TLR2(−/−) (TLR2KO) bone marrow (BM). Reciprocal radiation BM chimeras were generated as described in Results and Materials and Methods. Four types of chimeric mice, (i) TLR2KO BM transplanted into TLR2KO background, (ii) TLR2KO BM into WT background, (iii) WT BM into TLR2KO background, and (iv) WT BM into WT background, were injected sc with 20 µg/mouse sLP and irradiated with 9 Gy TBI 24 h later (*n = 8*/group). Survival was monitored for 30 days. (C) sLP-mediated cytokine induction in chimeric mice with WT or TLR2KO BM. The four types of chimeric mice described in (B) were injected with 20 µg/kg sLP without irradiation. Four sLP-injected mice of each genotype (2 males and 2 females) were euthanized at each time point (1, 2, 4, and 8 h post-injection) and serum cytokine levels of individual mice were determined using multiplex Luminex assays. The AUC for each cytokine was calculated as described in Materials and Methods and is presented for “TLR2KO to TLR2KO”, “TLR2KO-to-WT”, and “WT-to TLR2KO” chimeric mice as the percentage of that in “WT-to-WT” chimeric mice.

It is well documented that BLPs and their synthetic analogs interact with heterodimeric cell surface receptors comprised of TLR2 paired with either TLR6 or TLR1 and that this results in activation of NF-κB signaling [39], [40], [41]. We have shown that sLP treatment produces dose-dependent activation of NF-κB-dependent reporter gene expression in cultured cells expressing TLR2/6 or TLR2/CD14 receptor heterodimers, but not in TLR-negative cells (data not shown). To confirm that the *in vivo* radioprotective activity of sLP is dependent upon its interaction with TLR2 receptor complexes, we compared its effects in TLR2 knockout (KO) mice and isogenic wild type (WT) control C57BL/6J mice (see Materials and Methods). WT and TLR2 KO C57BL/6J mice were exposed to 9 Gy TBI 24 h after sc injection of PBS or sLP (3 µg/mouse). At 30-days post-irradiation, there were no surviving PBS-treated WT mice (0/15), confirming 9 Gy TBI as the LD_100/30_ for C57BL/6J mice (Figure 4A). In contrast, 100% (15/15) of WT mice that received sLP prior to 9 Gy TBI survived to day 30 post-irradiation. In TLR2 KO mice, sLP pre-treatment did not ameliorate radiation-induced death. Thirty-day survival was 0% (0/15) in the sLP-treated TLR2 KO group and 13% (2/15) in the corresponding PBS-treated group. These data clearly indicate that TLR2-containing receptor complexes are required for sLP-mediated radioprotection.

### Responses of both bone marrow and non-bone marrow cells contribute to sLP-mediated radioprotection and cytokine induction

Having determined that TLR2 expression is critical for sLP-mediated radioprotection/mitigation, we generated TLR2 KO/wild type chimeric mice to evaluate the roles of bone marrow (BM)-derived cells versus other cell types in this effect. Reciprocal BM mouse chimeras were generated as follows: TLR2 KO or wild type (WT) C57BL/6 “recipient” mice were lethally irradiated (2×6 Gy TBI 24 h apart) and then transplanted with BM cells (5×10^6^ cells/recipient mouse) from TLR2 KO or WT C57BL/6 “donor” mice (untreated and unirradiated). The level of chimerism in the transplanted mice was checked 60 days later by FACS analysis as described in Materials and Methods. Only chimeric mice with >95% of peripheral blood cells displaying surface antigens specific to the BM donor mouse strain were used in subsequent experiments.

Groups of 8 chimeric mice for each of the four types ((i) TLR2KO BM transplanted into TLR2KO background (“TLR2KO-to-TLR2KO”), (ii) TLR2KO BM into WT background (“TLR2KO-to-WT”), (iii) WT BM into TLR2KO background (“WT-to-TLR2KO”) and (iv) WT BM into WT background (“WT-to-WT)) were tested for sLP-mediated radioprotection. Mice were injected sc with 20 µg of sLP and then irradiated 24 h later with 9 Gy TBI. As expected based on our results with non-chimeric WT mice, “WT-to-WT” chimeras were protected from radiation-induced death by sLP pretreatment (88% 30-day survival, figure 4B). Similarly, WT mice transplanted with TLR2-negative BM cells (“TLR2KO-to-WT” chimeras) were fully protected (100%). On the other hand, mice completely deficient in TLR2 (“TLR2KO-to-TLR2KO” chimeras) were not protected (0% 30-day survival) and mice with TLR2 expression only in the BM (“WT-to-TLR2” chimeras) were only partially protected (50% survival). Our unrelated studies using another mouse strain (CD2F1) with BMT after lethal irradiation demonstrated that mice did not show altered sensitivity to TBI after they recovered from transplantation of syngeneic bone marrow (data not shown). These data show that TLR2 expression in BM cells is partly dispensable for sLP-mediated radioprotection, while TLR2 expression in non-BM cells is not.

We next treated the four established types of chimeric mice (“WT-to-WT”, “TLR2KO-to-WT,” “WT-to-TLR2KO” and “TLR2KO-to-TLR2KO”) with sLP in the absence of radiation to determine the contributions of BM- and non-BM cells to sLP-mediated cytokine induction. Twenty animals of each type of chimeric mice (10M+10F) were injected with 20 µg/kg of sLP. At time points 0 (before injection), 1 h, 2 h, 4 h, and 8 h after injections 4 mice/time point (2M and 2F) were euthanized, blood was collected and used to prepare serum for measurement of cytokine levels using multiplex Luminex assays. The Total Amount of Produced Cytokines (TAPC) was calculated as the sum of the amount of cytokine at all measured time points after sLP injection and averaged for the four animals in each group. These mean TAPC values are presented as a percentage of the mean value for the “WT-to-WT” chimeric group in Figure 4C. The level of G-CSF in the serum of sLP-treated “TLR2KO-to-WT” chimeric mice was similar to (89% of) that in “WT-to-WT” mice. However, sLP-mediated induction of G-CSF was much lower in “WT-to-TLR2KO” mice (4% of the “WT-to-WT” value) and essentially absent in “TLR2KO-to-TLR2KO” mice. These data confirm that sLP-mediated induction of G-CSF requires TLR2. However, since chimeric mice with TLR2-negative BM cells on a WT background showed the same G-CSF response to sLP as completely WT mice, it is clear that direct response of BM cells to sLP is not involved in the observed upregulation of G-CSF. Rather TLR2 expression in the mouse background (non-BM cells) is critical for G-CSF induction. All of the other tested cytokines (IL-1β, IL-6, IL-10, IL-12(p70), GM-CSF, KC, and TNF-α) showed a similar pattern of expression in the different chimeras: the level of induction was closest to “WT-to-WT” in “TLR2KO-to-WT” chimeric mice, reduced in “WT-to-TLR2KO” mice, and lowest in “TLR2KO-to-TLR2KO” animals. Like G-CSF, IL-6 and KC showed essentially no sLP-mediated induction in mice completely deficient in TLR2 (“TLR2KO-to-TLR2KO” chimeras), while the other tested cytokines showed residual induction (∼10–30% of “WT-to-WT). The contribution of BM was greater for sLP-induced cytokines other than G-CSF; however, in all cases it was clear that non-BM cells also play a role (TLR2 expression in the mouse background led to higher levels of cytokine induction).

Taken together, the results obtained with TLR2KO-WT chimeric mice demonstrate that the beneficial effect of sLP on the HP system following irradiation is at least partially indirect (i.e., not mediated by a direct response in radiosensitive BM cells). Additionally, the data suggest that (i) G-CSF is likely the main mediator of the radioprotective efficacy of sLP (since sLP specifically protects “TLR2KO-to-WT” chimeras and G-CSF shows a unique profile of induction in mice of this genotype), (ii) cytokines other than G-CSF might act to potentiate the effect of sLP on post-irradiation survival (since sLP partially protected “WT-to-TLR2KO” chimeric mice and there is very little G-CSF induction in this genotype, although other cytokines are induced), and (iii) the low levels of induction of other cytokines observed in sLP-treated “TLR2KO-to-TLR2KO” chimeras are not involved in protection of mice from radiation-induced death or at least are not sufficient to confer such protection.

## Discussion

This work demonstrates the radioprotective and radiomitigative capacity of sLP that mimic the N-terminal structure of naturally occurring mycoplasmal lipoproteins and their ability to activate NF-κB via TLR2-containing receptors. sLP is notable in that it significantly increases survival of lethally irradiated mice when administered as a single injection as early as 48 h before irradiation or as late as 24 h after irradiation. While sLP is effective as a radiomitigator injected after TBI, its efficacy is greater when administered before TBI. When injected at the optimal prophylactic time of 24 h before TBI, sLP increased 30-day survival of ICR mice exposed to 10 Gy TBI from 0% to 100% (Fig. 1). The DRF for sLP administered to ICR mice prior to irradiation was calculated to be 1.32. This indicates that the TBI dose needed to cause 50% lethality within 30 days was 1.32-fold higher in sLP-treated mice than in vehicle-treated mice. This DRF is in the same range as those for many other proposed radiation countermeasures (e.g., Vitamin E (α-tocopherol) with a DRF of 1.23 [42], 5-AED with a DRF of 1.26 [43], SCF with a DRF in the range of 1.3–1.35 [14], etc.). Nevertheless, there are examples of compounds with higher DRFs (e.g., amifostine with a DRF up to 2.0 in mice [21] and the flagellin derivative CBLB502 with a DRF of 1.6 [33]). It should be noted, however, that sLP has an important advantage over some other compounds in the context of prophylactic use since it is practically non-immunogenic. This overcomes the biggest problem associated with repeated administration of CBLB502.

Another important advantage of sLP is that, in addition to acting as a radioprotectant, it is also effective as a radiomitigator administered *after* radiation exposure has occurred. Athough post-irradiation treatment with sLP was not effective against 10 Gy TBI and the maximal increase in survival observed in ICR mice after 9 Gy TBI was 65% (Fig. 2), the observed radiomitigative efficacy of sLP is nevertheless a relatively unique feature among MRC under development and is clearly important for potential use in biodefense applications in which advance warning of radiation exposure is not likely to be available. Radiomitigative efficacy has also been demonstrated for vitamin E [44] and the TLR5 agonist CBLB502 [33], whereas amifostine is not effective when administered after exposure [45]. We speculate that the lower DMF (efficacy) observed with post-irradiation (mitigative) sLP treatment as compared to pre-irradiation treatment might be explained as follows: when applied after irradiation, the ability of a drug to suppress apoptosis in damaged cells can no longer contribute to its efficacy, only its ability to stimulate regeneration can. This hypothesis can be tested in future detailed studies of the efficacy and mechanism of action of sLPs as radiation countermeasures.

An additional feature of sLP treatment that can be noted as a benefit in terms of likely biodefense applications is that this agent is effective against high dose TBI delivered at a high or low dose rate (as tested in this study, within several minutes or over 60–70 hours, respectively). It is not clear whether other radiation countermeasures share this feature since low dose rates have not been tested in most previous studies. sLP also has the advantage of being efficacious as a single injected dose. While this characteristic is particularly important for biodefense applications, it provides a clear benefit even in medical scenarios over drugs such as G-CSF (Neupogen®) which requires multiple daily injections for up to 2 weeks in chemotherapy patients.

The radioprotective and radiomitigative effects of sLP are limited to doses of TBI that cause primarily HP syndrome-dependent mortality. While sLP's lack of efficacy against higher, GI syndrome-inducing TBI doses may be viewed as a disadvantage vis-à-vis countermeasures such as amifostine and CBLB502, HP-specific radiation countermeasures can be projected to have a significant impact in many biodefense and medical scenarios in which both short-term mortality and long-term health consequences stem from radiation damage to the HP system. In this communication, we report that sLP treatment accelerated regeneration of radiation-depleted bone marrow cells, spleen cells, and thrombocytes. Ongoing experiments are focused on more precisely defining the effects of sLP on different tissues and cell lineages of the HP system, such as hematopoietic stem cells.

In terms of mechanism of action, testing of sLP in TLR2 knockout (KO) mice confirmed that the ability of sLP to reduce the lethality of TBI is dependent upon TLR2. Moreover, through analysis of TLR2 KO/WT bone marrow chimeras, we showed that TLR2 responses to sLP in both BM and non-BM cells contribute to the radioprotective efficacy of this agent. This indicates that the beneficial effects of sLP on HP cells are mediated, at least in part, through indirect, non-cell autonomous mechanisms. The involvement of such indirect mechanisms is consistent with the capacity of sLP to reduce radiation damage even when administered after radiation exposure. Therefore, the anti-ARS activity of sLP likely involves multiple mechanisms including direct protection of radiosensitive cells (via activation of NF-κB-dependent anti-apoptotic factors) as well as indirect effects mediated by sLP-induced cytokines (see below) or other factors produced by BM-derived cells as well as cells outside the HP system. Such indirect effects might impact both preservation of HP tissue cellularity (protection against cell death) and stimulation of tissue regeneration.

Injection of sLP resulted in strong transient induction of a number of cytokines with known roles in hematopoiesis, including G-CSF, KC, and IL-6. Thus, sLP-induced cytokines are likely to mediate, at least in part, the radioprotective/mitigative activity of this countermeasure. In particular, G-CSF is a promising candidate mediator of sLP's radioprotective activity due to its striking induction following sLP injection (Fig. 3A) and its known biological effects. Recombinant G-CSF (Neupogen®) is currently widely used in the clinic to facilitate recovery of the HP system in situations such as bone marrow transplantation and chemotherapy. However, we found that a single injection of sLP was just as effective as 16 daily injections of Neupogen® in increasing survival of irradiated mice (Fig. S2B). The efficacy of sLP likely derives from its ability to not only induce G-CSF, but also multiple other cytokines that impact HP cell differentiation, proliferation, and survival. Therefore, sLP can be projected to be preferred as a radiation countermeasure over single cytokine therapies. It would be interesting to evaluate changes in cytokine levels when sLP is applied after irradiation to define the mechanisms responsible for the observed dependence of efficacy on the time of drug injection relative to irradiation. Notably, the TLR5 agonist CBLB502 produces a cytokine response that is very similar to that induced by sLP [33], but protection/mitigation of GI ARS is only seen with CBLB502, and not with sLP. This suggests that the induced cytokines are not involved in, or at least not sufficient for, altering the course of radiation-induced events in GI cells.

Overall, this study illustrates the strong potential of sLP-based TLR2 agonists for radioprotection and mitigation in defense and medical scenarios. The biodefense indication is key given the risk level in today's world and the fact that there are currently no FDA-approved radiation countermeasures suitable for use in mass-exposure scenarios [46]. Medical use of sLP for protection against cancer treatment side effects would also have a substantial impact on human health. However, development of sLP in this direction will require clarification of some critical issues. Foremost, does sLP protect not only normal cells, but also tumors, against radiation-induced killing? Although this remains to be tested directly for sLP, our previous work with the TLR5 agonist CBLB502 indicated that the anti-radiation effects following from activation of NF-κB via TLR stimulation were indeed specific to normal cells [33] and, therefore, not sensitive to the stimulatory effects of TLR agonists. Intratumoral administration in humans [47] or systemic administration in experimental tumor models in mice [48] demonstrated that sLP administration is not only safe during anti-cancer therapy, but provides survival benefits as well. Moreover, TLR-mediated NF-κB signaling is known to activate both the innate and adaptive immune systems, including anti-tumor immunity [49], [50], [51], [52]. Thus, temporary activation of NF-κB by sLP might not only result in radioprotection of normal tissues, but also reduce the incidence of secondary cancers due to the simultaneous immunostimulatory effect of NF-κB activation. Second, does sLP treatment have any positive effect on tumor growth or metastasis? This will be important to resolve given a recent report [53] indicating that TLR2 agonism can stimulate metastasis. Third, is sLP effective against radiation delivery characteristic of anti-cancer radiotherapy regimens (e.g., local fractioned irradiation)? This has also been positively resolved for the TLR5 agonist CBLB502 [54] and is readily testable for sLP. Ongoing work is focused on resolving these issues and developing optimized sLP for use as safe, practical and effective radiation countermeasures. The data herein provide a strong foundation for these efforts, demonstrating that sLP has a number of promising characteristics including a broad time window for effective administration, attractive DRF, efficacy against slowly delivered radiation, and capacity to induce cytokines with desirable activities.

## Materials and Methods

### Mice

Female 10–12 week-old ICR(CD1) mice were purchased from Harlan (Indianapolis, IN). Female 10–12 week-old BALB/cJ, C57BL/6J, B6.SJL-Ptprc^a^ Pepc^b^/BoyJ, and breeding pairs of TLR2(−/−) mice (C57BL/6 genetic background after 11 backcrosses) were purchased from the Jackson Laboratory (Bar Harbor, ME). All mice were housed (up to 5 mice per cage with exception of ICR - 4 mice per cage) in an air-conditioned facility accredited by the Association for Assessment and Accreditation of Laboratory Animal Care International. All mice were maintained in rooms on a 12-h light/dark cycle, at 21±2°C, with 10–15 hourly cycles of fresh air, and relative humidity of 50±10%. Upon arrival, the mice were held in quarantine for 1 week and provided certified rodent rations and acidified water (HCl, pH = 2.5–2.8) *ad libitum*. All animal procedures were performed according to protocols approved by Institutional Animal Care and Use Committees of the Cleveland Clinic Foundation (CCF), Roswell Park Cancer Institute (RPCI), and Indiana University. Research was conducted according to the Guide for the Care and Use of Laboratory Animals prepared by the Institute of Laboratory Animal Resources, National Research Council, U.S. National Academy of Sciences.

### Irradiation

For all experiments except those shown in Figures 2C and S2B (described below), mice were exposed to bilateral total body irradiation (TBI) using J.L. Shepherd MK I-68 ^137^Cs γ-irradiators located at Cleveland Clinic Foundation (dose rate = 2.3 Gy/min on the day of irradiation) or Roswell Park Cancer Institute (dose rate = 1.55 Gy/min on the day of irradiation). Dose rates were recalculated daily. Mice were irradiated in a well-ventilated Plexiglas bucket accommodating 8–10 mice per irradiation round. The irradiation bucket was elevated on the irradiator turntable using a 2 cm-high plastic riser in order to provide a more uniform radiation field. For irradiation, mice from different treatment groups were mixed and then returned to their corresponding cages.

Experiments shown in Figure 2C (low dose rate irradiation) were conducted at Colorado State University (Fort Collins, CO) using a J.L. Shepherd Model 81 S/N 7014 ^137^Cs (600Ci) γ-irradiator with a dose rate 0.41±0.05 cGy/min under CSU IACUC protocol #07 - 239A. Sustained irradiation for 60 or 70 hours provided total irradiation doses of 14.4 Gy and 16.8 Gy, respectively.

The experiment shown in Figure S2B was performed at the Indiana University School of Medicine (Indianapolis, IN). Groups of C57BL/6 mice were irradiated using a GammaCell 40 ^137^Cs γ-irradiator (Nordion International, Kanata, Ontario, Canada) with a dose rate of 675 cGy/min to achieve a total dose 7.96 Gy.

### Reagents

R-Pam_2_-CSKKKK (*S*-[(2*R*)-2,3-bis(palmitoyloxy)propyl]-cysteinyl-SKKK) and other sLP were purchased from EMC microcollections GmbH (Tuebingen, Germany) as pure dry powder. The powder was reconstituted in D-PBS at a concentration of 2.5 mg/ml and stored as a stock solution for up to 6 months at +4°C. Final desired dilutions were made in D-PBS just before administration. Each vial of Neupogen® (Amgen, Inc., Thousand Oaks, CA) contained 300 µg of r-metHuGCSF (at a specific activity of 1.0±0.6×108 Units/mg; 30 million total units/vial) in a total volume of 1 ml. Neupogen® was stored at 4°C and administered to mice at a dose of 2.5 µg/mouse/day (∼125 µg/kg/day dose based on expected average mouse weight of 20 g; actual average mouse weights were 15–21.5 g and 19–28 g for females and males, respectively, on the day before TBI). All substances were administered to mice via subcutaneous or intraperitoneal injections without anesthesia.

### Preparation of WT and TLR5KO bone marrow cells for generation of bone marrow chimeras

Preparation of bone marrow single cell suspensions was performed as previously described [55]. Briefly, 4 bones per mouse (2 tibias and femurs) from 3 donor mice of each genotype were flushed with 1 ml medium (Iscove's +0.5% BSA, no antibiotics) into 5 ml round-bottomed tubes. Cell suspensions were filtered through a 40 µm strainer into a 50 ml tube. Washed cells were collected by centrifugation and resuspended in 2.0 ml medium for counting of nucleated cells. After counting, the volume was adjusted to have ∼5×10^6^ cells/recipient in 0.2 ml volume.

### Preparation of spleen cells

Single cell suspensions from spleens were prepared as described [56]. Viable (trypan blue-excluding) cells were counted under a microscope.

### Preparation of mouse bone marrow (BM) radiation chimeras

Four types of reciprocal radiation BM chimeras were preapred: TLR2 (−/−) mice reconstituted with either TLR2 (−/−) or wild type (WT) BM cells and WT mice reconstituted with either TLR2 (−/−) or WT BM cells. B6.SJL-Ptprc^a^ Pepc^b^/BoyJ (CD45.1) mice were used as the WT counterpart to TLR2 (−/−) mice on a C57BL/6 (CD45.2) genetic background. Recipient mice were exposed to two rounds of TBI (6 Gy/round) 24 h apart. Within 3 hours after the second dose of TBI, recipient mice were injected iv with 5×10^6^ BM cells prepared from unirradiated donor mice of the appropriate genotype. Sixty days after BM transplantation, 100 ∝l of blood was obtained from the tail vein of recipient mice and analyzed by FACS to determine the extent of chimerism in each mouse. For FACS analysis, samples of whole blood were stained with the following antibodies (2 ∝l/10^6^ cells): CD45.1 FITC-labeled (11-0453-82), Ly-6G PE-labeled (12-5931-85), CD19 PE-Cy5.5-labeled (35-0193-82), and CD45.2 APC-labeled (17-0454-82) (eBioscience, San Diego, CA 92121). After 30 min staining, red blood cells were lysed by adding 1 ml pre-warmed (37°C) lysis buffer (00-4333-57, eBioscience) and mixing at room temperature for 5 min. White blood cells were collected by centrifugation at 400 g for 10 min at 4°C, resuspended in 300 µl staining buffer (00-4222-57, eBioscience) and analyzed using a four-color FACSCalibur instrument (Becton Dickinson, San Diego, CA). BM chimeric mice were used in experiments only if >98% of GR1^+^ and CD11b^+^ cells were donor-derived (positive for CD45.1 or CD45.2 as appropriate depending upon donor genotype) and if >90% of CD19^+^ and CD3^+^ cells were donor derived.

### Analysis of serum cytokine levels

For cytokine analysis, blood was collected 1, 2, 4, 8, 24, or 48 h after sLP injection. Blood was collected from mice by cardiac puncture, transferred to CapiJect serum separator tubes (T-MQK, TERUMO, Somerset, NJ), and centrifuged at 1000 rpm for 10 min. Serum was stored at −70°C until used for cytokine analysis.

Serum levels of multiple cytokines were simultaneously determined using cytokine analysis kits custom ordered (M200003JZX) from Bio-Rad, Inc. (Hercules, CA) on with the Luminex-200 dual-laser flow analyzer (Luminex Corp, Austin, TX). Interleukin-1β (IL-1β), IL-6, IL-10, IL-12(p70), granulocyte colony-stimulating factor (G-CSF), granulocyte macrophage colony-stimulating factor (GM-CSF), keratinocyte-derived chemokine (KC), and tumor necrosis factor-α (TNF-α) were measured. The bead-based sandwich immunoassay kits from Bio-Rad included all necessary reagents for cytokine analysis. Briefly, anti-cytokine antibody-conjugated beads were added to wells of flat-bottom 96-well plates (Bio-Rad, Inc.). Serum samples diluted 1∶4 with the provided diluent were added to the wells. After incubation, plates were washed using a Bio-Plex Pro wash station (Bio-Rad, Inc.). Diluted detection antibody was added and plates were incubated for 1 h. After washing again, streptavidin-phycoerythrin was added. After final incubation and washing, the signal from the bound fluorochrome was quantified using the Luminex-200 analyzer. The instrument was calibrated with calibration microspheres (Bio-Rad, Inc.). The median fluorescence intensity of fluorochrome-conjugated antibody bound to individual microspheres was derived from flow analysis of 50 microspheres/region. For quantification of cytokines, standard curves were plotted using standards supplied with kit. The intensity of the fluorescence was directly proportional to the concentration of cytokine. Calculations were performed using Bio-Plex Manger software version 5.0 (Bio-Rad, Inc.). The Total Amount of Produced Cytokines (TAPC) was calculated as the sum of the amount of cytokine at all measured time points after sLP injection.

### Statistical analysis

For survival experiments, the Log-Rank test was used to compare the kinetics of mortality (mean survival time of decedents). Fisher's Exact test was used to compare survival rates at the end of 30 days post-irradiation, with Bonferroni correction used to control for type-I errors if multiple comparisons were used. Results were considered statistically significant if *p*<0.05. All statistical tests were two-sided. For calculation of does modification factors (DRFs), Probit analysis was performed using the SPSS statistical package (http://spss.en.softonic.com/).

## Supporting Information

Figure S1 **Effect of sLP pre-treatment on survival of mice exposed to different doses of TBI.** Thirty-day Kaplan-Meier survival curves for groups of female ICR (CD-1®) mice injected sc with vehicle (PBS, *n = 14*) (A) or 40 µg/kg sLP (*n = 15*) (B) 24 hours before TBI with the indicated doses. TBI doses ranging from 7 Gy to 10 Gy for vehicle-treated groups (A) and from 10 to 13 Gy for sLP-treated groups (B) were used to cover LD_0–100/30_ lethality ranges.
(TIF)

Figure S2 **Effect of post-irradiation administration of sLP on survival of mice exposed to different doses of TBI.** (A) Thirty-day Kaplan-Meier survival curves for groups of female ICR (CD-1®) mice irradiated with the indicated TBI doses (7.5 - 10 Gy) and injected sc with 50 µg/mouse sLP 1 hour after TBI (*n = 15*/group). (B) Comparison of sLP and Neupogen® radiomitigation capacities. Groups of 30 C57BL/6 mice (15 males+15 females) were irradiated with 7.96 Gy TBI. Following irradiation, groups were treated as follows: (i) single sc injection of 50 µg/mouse (∼2.5 mg/kg dose based on expected average mouse weight of 20 g) sLP 3 hours after TBI; (ii) single sc injection of 50 µg/mouse sLP 24 hours after TBI; (iii) daily sc injection of Neupogen® starting 24 hours after TBI and continuing for 16 days at a dose of 2.5 µg/mouse/day (∼125 µg/kg/day dose based on expected average mouse weight of 20 g); and (iv) single sc injection of vehicle (PBS) at 24 hours after TBI. All groups contained 15 male and 15 female mice each, except for the Neupogen®-treated group which contained 15 males and 11 females. Mouse survival was monitored for 30 days. Fisher's Exact tests were used to determine whether differences in 30-day survival were statistically significant.
(TIF)

Figure S3 **Effect of sLP pre-treatment on radiation-induced changes in mouse hematopoietic organs.** Female BALB/c mice (n = 24/group) were injected ip with vehicle (PBS) or sLP (3 µg/mouse) 24 h before exposure to 4 Gy TBI. Bone marrow (from two femurs of each individual mouse) (A) and spleen (B) cell suspensions were prepared (3 ml final volume) from 8 mice euthanized on days 1, 7 and 14 after TBI. Bone marrow and spleen cell suspensions were prepared similarly from 8 age-matched naïve control mice were not injected or irradiated. Viable (trypan blue-excluding) cells were counted under a microscope. Error bars indicate standard errors.
(TIF)
