# Supplementary figures and images for: Prevention and Mitigation of Acute Radiation Syndrome in Mice by Synthetic Lipopeptide Agonists of Toll-Like Receptor 2 (TLR2)
Source: PLoS One. 2012 Mar 27;7(3):e33044. doi: 10.1371/journal.pone.0033044 (PMC3314012; doi:10.1371/journal.pone.0033044)

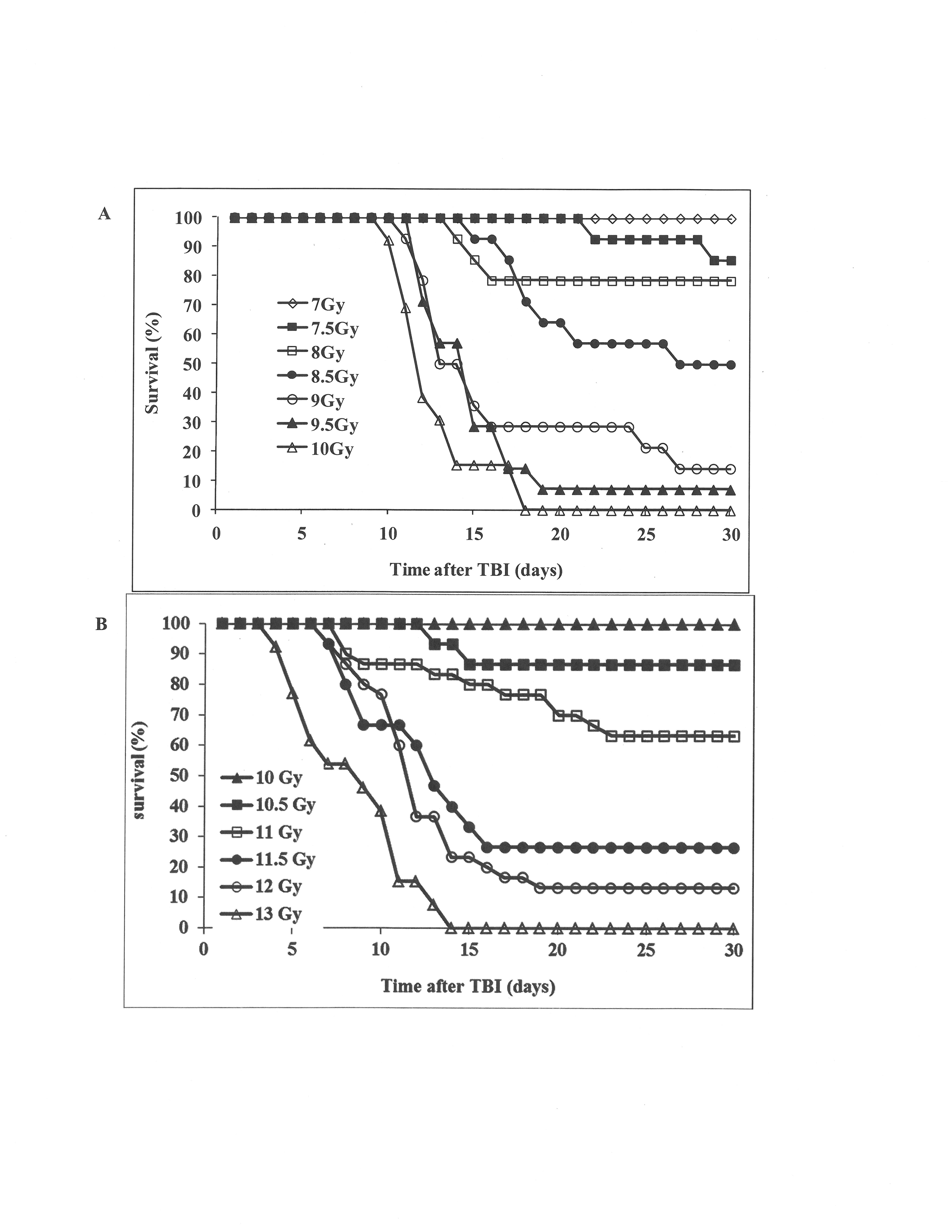

Supplement: Figure S1 — Effect of sLP pre-treatment on survival of mice exposed to different doses of TBI. Thirty-day Kaplan-Meier survival curves for groups of female ICR (CD-1®) mice injected sc with vehicle (PBS, n = 14) (A) or 40 µg/kg sLP (n = 15) (B) 24 hours before TBI with the indicated doses. TBI doses ranging from 7 Gy to 10 Gy for vehicle-treated groups (A) and from 10 to 13 Gy for sLP-treated groups (B) were used to cover LD0–100/30 lethality ranges. (TIF) [file pone.0033044.s001.tif]

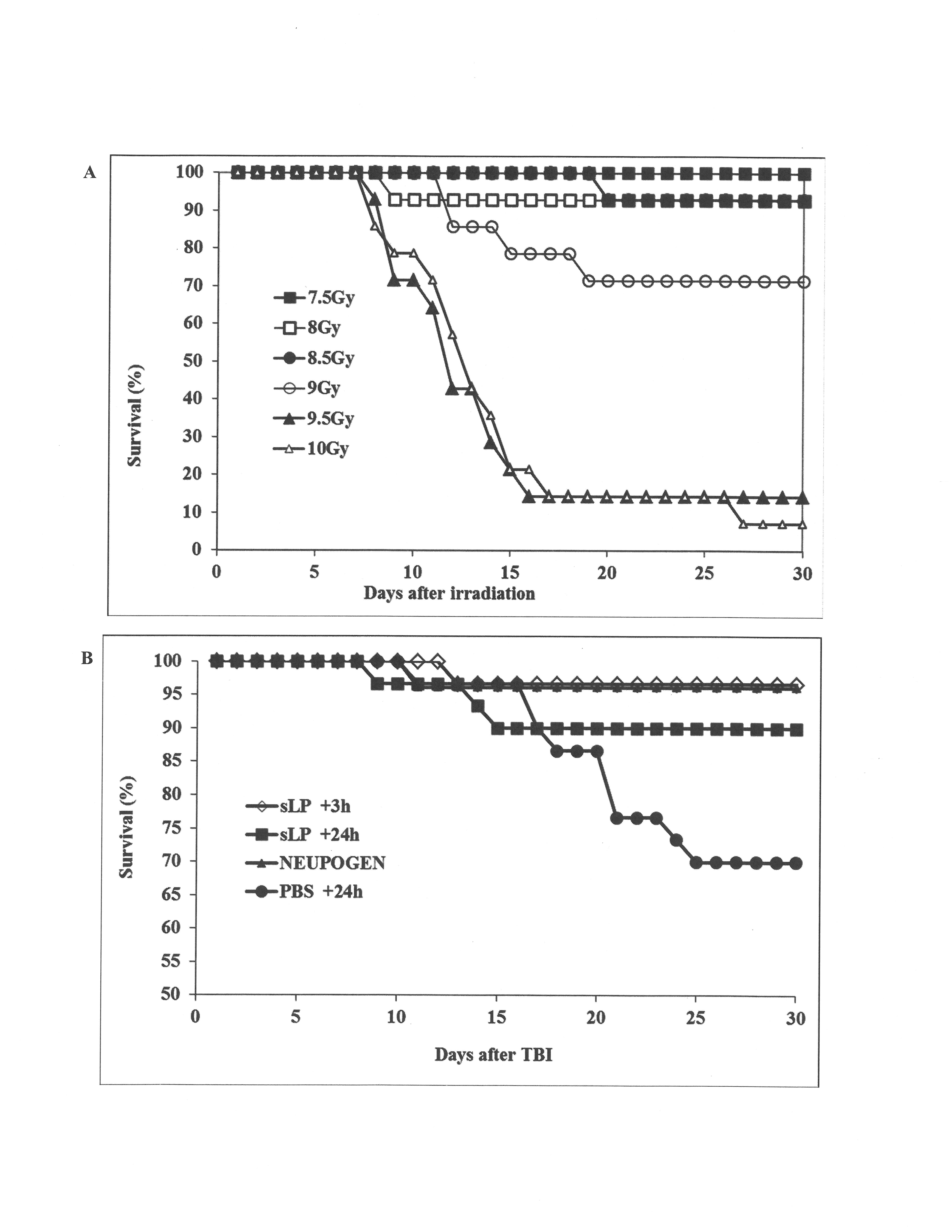

Supplement: Figure S2 — Effect of post-irradiation administration of sLP on survival of mice exposed to different doses of TBI. (A) Thirty-day Kaplan-Meier survival curves for groups of female ICR (CD-1®) mice irradiated with the indicated TBI doses (7.5 - 10 Gy) and injected sc with 50 µg/mouse sLP 1 hour after TBI (n = 15/group). (B) Comparison of sLP and Neupogen® radiomitigation capacities. Groups of 30 C57BL/6 mice (15 males+15 females) were irradiated with 7.96 Gy TBI. Following irradiation, groups were treated as follows: (i) single sc injection of 50 µg/mouse (∼2.5 mg/kg dose based on expected average mouse weight of 20 g) sLP 3 hours after TBI; (ii) single sc injection of 50 µg/mouse sLP 24 hours after TBI; (iii) daily sc injection of Neupogen® starting 24 hours after TBI and continuing for 16 days at a dose of 2.5 µg/mouse/day (∼125 µg/kg/day dose based on expected average mouse weight of 20 g); and (iv) single sc injection of vehicle (PBS) at 24 hours after TBI. All groups contained 15 male and 15 female mice each, except for the Neupogen®-treated group which contained 15 males and 11 females. Mouse survival was monitored for 30 days. Fisher's Exact tests were used to determine whether differences in 30-day survival were statistically significant. (TIF) [file pone.0033044.s002.tif]
